# Texas Public Agencies’ Tweets and Public Engagement During the COVID-19 Pandemic: Natural Language Processing Approach

**DOI:** 10.2196/26720

**Published:** 2021-04-26

**Authors:** Lu Tang, Wenlin Liu, Benjamin Thomas, Hong Thoai Nga Tran, Wenxue Zou, Xueying Zhang, Degui Zhi

**Affiliations:** 1 Department of Communication Texas A&M University College Station, TX United States; 2 Jack J Valenti School of Communication University of Houston Houston, TX United States; 3 Department of Computer Science Rice University Houston, TX United States; 4 School of Biomedical Informatics University of Texas Health Science Center at Houston Houston, TX United States; 5 Department of Journalism and Mass Communication North Carolina A&T State University Greensboro, NC United States

**Keywords:** COVID-19, public health agencies, natural language processing, Twitter, health belief model, public engagement, social media, belief, public health, engagement, communication, strategy, content analysis, dissemination

## Abstract

**Background:**

The ongoing COVID-19 pandemic is characterized by different morbidity and mortality rates across different states, cities, rural areas, and diverse neighborhoods. The absence of a national strategy for battling the pandemic also leaves state and local governments responsible for creating their own response strategies and policies.

**Objective:**

This study examines the content of COVID-19–related tweets posted by public health agencies in Texas and how content characteristics can predict the level of public engagement.

**Methods:**

All COVID-19–related tweets (N=7269) posted by Texas public agencies during the first 6 months of 2020 were classified in terms of each tweet’s functions (whether the tweet provides information, promotes action, or builds community), the preventative measures mentioned, and the health beliefs discussed, by using natural language processing. Hierarchical linear regressions were conducted to explore how tweet content predicted public engagement.

**Results:**

The information function was the most prominent function, followed by the action or community functions. Beliefs regarding susceptibility, severity, and benefits were the most frequently covered health beliefs. Tweets that served the information or action functions were more likely to be retweeted, while tweets that served the action and community functions were more likely to be liked. Tweets that provided susceptibility information resulted in the most public engagement in terms of the number of retweets and likes.

**Conclusions:**

Public health agencies should continue to use Twitter to disseminate information, promote action, and build communities. They need to improve their strategies for designing social media messages about the benefits of disease prevention behaviors and audiences’ self-efficacy.

## Introduction

COVID-19 is a new infectious disease that is caused by SARS-CoV-2, a new and potentially deadly coronavirus. The ongoing COVID-19 pandemic is characterized by different morbidity and mortality rates across different states, cities, rural areas, and diverse neighborhoods. The absence of a national strategy for battling the pandemic also leaves state and local governments responsible for creating their own response strategies and policies [1]. However, misinformation and disinformation continue to circulate on social media platforms with unprecedented volume and velocity, which affects the public’s trust in and response to governmental restrictions and corrective actions [2,3]. Thus, it is crucial to examine how state and local health departments communicate with their stakeholders on social media platforms.

Public health agencies have been actively using platforms such as Twitter and Facebook to communicate with their stakeholders during public health crises. The accumulating literature on organizational social media use has identified the following three primary functions: information, action, and community [4]. The information function refers to the organizational use of social media to provide the public with emergency and risk information [5]. It includes a wide range of activities such as making emergency updates, advisories, and warnings; providing scientific explanations and public education; and clarifying misinformation about an unfolding epidemic [6]. The action function refers to how organizations can use social media to encourage their followers to adopt or avoid certain behaviors [4], such as attending events, making monetary donations, volunteering, and adopting other recommended behaviors. In the context of health and risk communication, action-oriented messages may specify how individuals can protect themselves when an imminent threat arises, and this function is directly related to the overarching goal of public health agencies, which is to mitigate risk behaviors during an epidemic [7]. The community function revolves around building relationships with community members, providing social and emotional support, and communicating about collective identities. Providing emotional support and boosting community morale can enhance public trust and cooperative behaviors [8], of which both are essential for effective risk mitigation. Although health agencies are generally advised to use multiple social media functions, such as those outlined above, a large body of earlier studies has suggested that most public agencies’ social media messages are disseminated via one-way communication [9]. Thus, we propose the first research question (RQ) about the functions of public agencies’ tweets during the COVID-19 pandemic, as follows: to what extent do public health agencies’ Twitter messages fulfill the functions of information, action, and community during the COVID-19 pandemic (RQ1)?

According to the Health Belief Model (HBM), a person’s decision to adopt a recommended health behavior is influenced by their desire to avoid an illness and their belief that the recommended behavior can help prevent an illness [10]. The following two factors affect one’s desire to avoid an illness: perceived severity and perceived susceptibility. When a person thinks that an illness is serious (perceived severity) and that they have a high chance of contracting it (perceived susceptibility), they will be more alarmed and want to avoid the illness. Further, an individual’s preventative behavior is also influenced by their beliefs about (1) whether the recommended behavior can indeed provide health benefits, such as preventing the illness (perceived benefits); (2) the obstacles associated with adopting the recommended behavior, such as cost and time (perceived barriers); and (3) their ability to engage in the behavior (self-efficacy). A meta-analysis study on the decades of research that involved the use of the HBM has indicated that perceived benefits and perceived barriers are the strongest predictors of behavioral change [11].

The original HBM was a psychological model that was created to predict an individual’s health behaviors. It has recently been used to guide the design of health messages for effectively promoting health behaviors and evaluate the presence or absence of elements in media content that might contribute to people’s health beliefs [12]. Understanding the extent to which public health agencies’ tweets address different health beliefs could offer insights into how these tweets might inform the public about the threats of COVID-19 and encourage proper preventative measures. Hence, we propose the next set of RQs, as follows: (1) what are the recommended preventative behaviors (RQ2a) and (2) to what extent do public health agencies communicate severity, susceptibility, benefits, barriers, and self-efficacy information in their Twitter messages about COVID-19 (RQ2b)?

In addition to behavioral outcomes, public engagement is another indicator of the effectiveness of public agencies’ crisis communication efforts. Public engagement refers to the various forms of communicative interaction between the public and government agencies, such as the public sharing or replying to governmental agencies’ messages [13]. Public engagement has several benefits. First, greater public engagement with public health agencies’ social media content typically indicates higher levels of exposure to, attention toward, and information absorption of the content in messages (eg, advisories, warnings, or other educational materials), which are essential in helping the public form accurate risk perceptions and encouraging risk-reduction behaviors [14]. Second, public engagement can be an indicator or precursor of trust in health institutions, which leads to better health adherence and other positive behavioral changes [15]. Finally, public engagement can help public health agencies identify, clarify, and correct misinformation, resulting in more effective health promotion [16]. Although public engagement is generally associated with positive outcomes, it should be noted that scholars distinguish between positive and negative engagement, suggesting that the latter may lead to the “denial, rejection, avoidance and negative word-of-mouth” of an organization [17]. For example, in the context of a crisis, it has been found that certain types of engagement may generate misinformation and undermine the authority of crisis management agencies [18].

We adopted Johnson and Taylor’s [19] conceptualization of public engagement at the individual level, which they defined as the public’s psychological and behavioral involvement and participation with public health agencies’ messages. In social media–mediated crisis communication, such individual-level engagement manifests in two forms: the public’s resharing behavior on social media [19,20] and the behavior of “liking” or endorsing public agencies’ social media messages. The first form of engagement (ie, sharing public agencies’ social media content with one’s own social networks) is viewed as an important outcome of effective health risk communication. Individuals’ sharing behavior on social media is a key mechanism that enables the amplification of public health agencies’ messages [21]. By sharing these messages via functions such as retweets, the public not only relays relevant health content to their immediate communities but also promotes collective sharing behaviors that can generate normative influences, which results in intended behavioral changes [22]. Additionally, it has been determined that endorsing public health agencies’ messages through Twitter’s “favorite” function or Facebook’s “like” function is a form of public engagement that is distinct from resharing [23,24]. Specifically, this endorsement behavior has been conceptualized as a type of affective engagement that indicates the audience’s feelings of support for or symbolic alignment with an organization with regard to a specific issue [25]. Although endorsement does not fully equate to the psychological acceptance of a message, research suggests that positive assessments are significantly associated with health message acceptance, especially when such endorsements are made by celebrities [26]. We thus propose the following question: how do the features of tweets predict public engagement in terms of the number of favorites and retweets during the COVID-19 pandemic (RQ3)?

## Methods

### Sampling and Data Collection

This study focused on public agencies in the state of Texas. Texas was chosen because this state became one of the disease epicenters following the enforcement of Governor Abbott’s state reopening measures in April 2020. At the time of data collection (mid-July 2020), Texas was facing the second peak of COVID-19 cases and had the highest 7-day average number of daily new cases (n=15,038) [27]. In addition, with Texas being the second largest and second most populous state in the United States, its public agencies may face the particularly challenging task of reaching out to the diverse population and coordinating with peer agencies. Since this study examined the public tweets of governmental agencies, it was exempt from human subjects ethics review.

We conducted the following steps to select the sample tweets for analysis. First, we identified all of the active Twitter accounts of public health departments and Office of Emergency Management (OEM) organizations at the city, county, and state levels in Texas. To identify public health departments, we obtained a list of health department directories from the Centers for Disease Control and Prevention and the US Department of Health and Human Services. Additionally, a list of local-level health agencies was obtained from the National Association of County and City Health Officials. In this step, we identified a total of 26 Texas public health departments that actively tweeted during the studied period. We also used a list of Texas city and county names to conduct searches on Twitter and identified an additional 56 official OEM organization Twitter accounts, which yielded a total of 82 organizations. Second, we created a list of 25 COVID-19–related keywords (“covid,” “corona,” “koronavirus,” “ncov,” “sars,” “pandemic,” “epidemic,” “quarantine,” “outbreak,” “handwash,” “wuhan,” “panic,” “chinese virus,” “lock down,” “sheltering in place,” “shelter in place,” “flatten the curve,” “safer at home,” “stay home,” “face covering,” “wear mask,” “get tested,” “quarantine,” “ppe,” and “n95”). All tweets from the 82 organizations that contained at least one of these keywords and were published between January 1 and June 30, 2020, were downloaded using Twitter’s developer application programming interface (n*=*15,382).

### Measurements

A codebook was developed to guide the coding of the training data set. It included the following variables: functions, types of recommended actions, and HBM variables. Each tweet was coded in terms of the presence or absence of COVID-19–related content. Tweets that contained COVID-19–related content were further coded.

First, each tweet was coded in terms of the functions it served. Tweets served the information function if they shared information about COVID-19, such as COVID-19 symptoms, risks of the disease, prevention information, current infection rates or case numbers, and testing information, or if they described actions that agencies were taking to contain COVID-19 spread. Tweets served the action function if they urged readers to adopt a certain health behavior. Tweets served the community function if they built community by asking readers to interact with each other and with the sender, providing emotional support, and boosting morale. These descriptions of functions were adapted from Kang [25]. Each tweet was evaluated in terms of whether it contained any of these three types of information.

Second, each tweet was coded in terms of whether it included one or more of the following actions: (1) handwashing, (2) social distancing, (3) mask wearing or face covering, (4) staying at home or sheltering in place, (5) getting tested, (6) learning more information, and (7) other behaviors.

Finally, HBM variables, including severity (any reference to the magnitude and seriousness of COVID-19), susceptibility (the likelihood that a person, a group, or the public in general will contract COVID-19), benefits (the benefits of recommended behaviors and their effectiveness in preventing or treating COVID-19 or containing the pandemic on the societal level), barriers (the difficulties associated with adopting or implementing the recommended behaviors), and self-efficacy (one’s ability to engage in recommended behaviors) were coded. TThe coding of these health beliefs was adapted from Tang and Park [12], respectively).

### Development of the Training Data Set

Several rounds of training sessions were conducted to assist two coders with understanding each item in the codebook. Afterward, around 20% (3000/15,000) of the tweets were used for the development of a training data set. Two coders coded 150 tweets that were randomly selected from the remaining 80% (12,000/15,000) of the tweets. These tweets achieved satisfactory intercoder reliability (Cohen κ: mean 0.83; range 0.56-0.96). Two items (barriers and self-efficacy) were dropped from the codebook because they were nearly completely absent from the collected tweets. Afterward, each coder independently coded half of the training data set.

### Computer-Assisted Classification Based on Natural Language Processing

Data cleaning was conducted by following the steps laid out by Du et al [28]. The bidirectional encoder representations from transformers (BERT), a natural language processing program developed by Google, was trained to automatically classify tweets [29]. The pretrained BERT-large model from Huggingface was used [30]. We divided the initial, manually coded data sets (3000 tweets) into a training data set (number of tweets: 2400/3000, 80%) and a testing data set (number of tweets: 600/3000, 20%). In our training set, some labels had a relatively low frequency (<250 occurrences), which resulted in these labels being mostly ignored in the model’s training process. To train such low-frequency categories, we doubled all instances of tweets with minority labels to give them a stronger signal in the model. The model was trained for 3 epochs by using the AdamW optimizer with a learning rate of 2e − 5.

Precision, recall, and overall F1 score (the harmonic mean of precision and recall) were calculated for each variable. We also calculated the microaveraging F1 score and macroaveraging F1 score to evaluate variables’ performance in each classification task. We summed up all of the individual true positives, false positives, and false negatives for the microaveraged score. For the macroaveraged score, we used the average of the F1 scores of different categories. Overall, our model achieved good results (Table 1). Afterward, we used the program to automatically classify all of the tweets in the sample.

**Table 1:** Bidirectional encoder representations from transformers classification of the performance of tweets about the COVID-19 pandemic that were published by Texas public health agencies between January 1 and June 30, 2020.

Variables	Precision^a^	Recall^b^	F1 score^c^
About COVID-19 or not	.93	.93	.93
Information function	.85	.92	.88
Action function	.68	.83	.75
Community function	.58	.58	.58
Handwashing	.75	1.00	.53
Social distancing	.80	.80	.80
Mask wearing or face covering	.85	.96	.90
Staying at home or sheltering in place	.74	.78	.76
Getting tested	.69	.90	.78
Learning more information	.77	.92	.84
Other behaviors	.27	.54	.36
Severity	.69	.92	.79
Susceptibility	.84	.86	.85
Benefit	.42	.70	.52

^a^The microaverage, macroaverage, weighted average, and sample average precision scores were .78, .70, .80, and .46, respectively.

^b^The microaverage, macroaverage, weighted average, and sample average recall scores were .88, .83, .88, and .50, respectively.

^c^The microaverage, macroaverage, weighted average, and sample average F1 scores were .83, .76, .84, and .47, respectively.

### Data Analysis

Hierarchical linear regressions or stepwise linear regressions were used to answer RQ3 (ie, how various tweet features predicted the numbers of favorites and retweets). This method enabled the assessment of separate effects from different blocks of variables. Since both variables for measuring engagement were highly skewed, we adopted the standard practice of log-transforming these metrics before they were entered into regression models. In the two regression models, the independent variables consisted of the following three blocks: (1) the information, action, and community message types; (2) the dichotomous thematic categories, which included social distancing, face covering, sheltering in place, getting tested, information seeking, and other behaviors; and (3) the health belief variables, which included severity, susceptibility, and benefits. To control for the effect of account popularity (popular accounts were more likely to promote greater public engagement), we entered the log-transformed number of followers as a control variable in each model.

## Results

A total of 7269 tweets were related to COVID-19. Of the 82 public health and OEM agencies, only 61 tweeted about COVID-19. These organizations tweeted about COVID-19 for an average of 119 times (SD 203.09).

RQ1 asked about the functions of tweets. Sharing information was the most prominent function of the tweets posted by public health agencies (6835/7269, 94.03%), followed by the action function (2491/7269, 34.27%). Community building was the least salient function, as only 10.19% (741/7269) of the tweets promoted the engagement of community members and provided emotional support.

**Figure 1:**
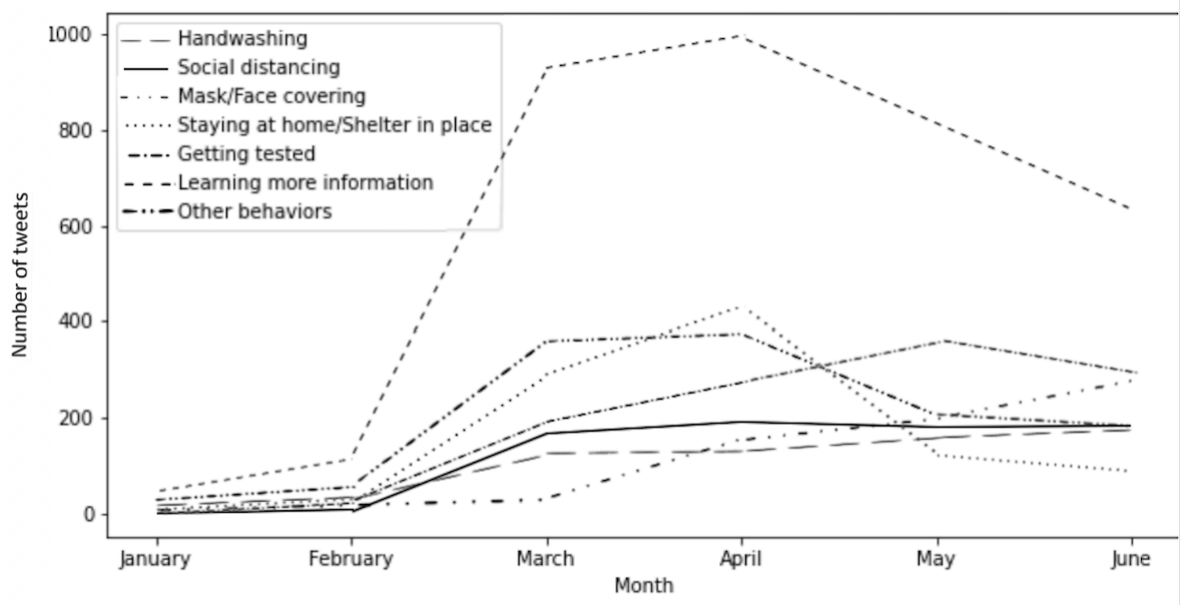
Longitudinal changes in the number of tweets promoting different health behaviors by public health agencies in Texas (January 1 to June 30, 2020).

RQ2 asked about the types of actions that tweets promoted and the health beliefs that tweets mentioned. Of the behaviors recommended by agencies, learning more information was the most recommended action among the tweets (3402/7269, 46.80%), followed by getting tested (1076/7269, 14.80%), staying at home or sheltering in place (911//7269, 12.53%), social distancing (700/7269, 9.63%), face covering (651/7269, 8.96%), and handwashing (616/7269, 8.47%). Figure 1 shows the number of tweets from public health agencies that mentioned different health behaviors over time. Handwashing was initially the most frequently recommended behavior, and its importance was continuously emphasized. Tweets that promoted staying at home or sheltering in place exhibited the sharpest increase in incidence, which dropped precipitously after April. Tweets that mentioned the action of getting tested increased in incidence between February and May but decreased in incidence during May and June. The number of tweets that mentioned social distancing started to plateau in March. The number of tweets that discussed the wearing of face coverings was minimal in the first 3 months, but this number started to consistently increase in March. In terms of HBM variables, severity (1389/7269, 19.11%), susceptibility (2057/7269, 28.30%), and benefits (1238/7269, 17.03%) were the three concepts that were frequently mentioned in public health agencies’ tweets.

RQ3 was proposed to examine the relationship between the content of tweets and public engagement. Overall, the public’s engagement with the tweets posted by public health agencies was relatively low, as each tweet had an average of 13.05 retweets (SD 43.16) and 19 favorites/likes (SD 59.97). Tables 2 and 3 present the two hierarchical regression models for predicting the two public engagement variables.

**Table 2:** Hierarchical ordinary least squares regression of predictors for the number of retweets (based on Texas public health agencies’ COVID-19–related tweets that were posted between January 1 and June 30, 2020; N=7269).

Variables		Number of retweets							
		Model 1^a^			Model 2^b^			Model 3^c^	
		β^d^ (SE)	*P* value	β (SE)		*P* value	β (SE)		*P* value
**Control**									
	Followers	.43 (.01)	<.001	.43 (.01)		<.001	.50 (.01)		<.001
**Functions**									
	Information	.10 (.03)	<.001	.09 (.02)		<.001	.04 (.03)		<.001
	Action	.10 (.01)	<.001	.09 (.01)		<.001	.12 (.02)		<.001
	Community	.01 (.02)	.35	.02 (.01)		.07	.04 (.02)		.001
**Types of actions proposed**									
	Handwashing	N/A^e^	N/A	−.01 (.03)		.36	−.05 (.03)		<.001
	Social distancing	N/A	N/A	.02 (.03)		.13	.02 (.03)		.16
	Mask wearing or face covering	N/A	N/A	.01 (.03)		.64	.03 (.03)		.01
	Staying at home	N/A	N/A	.05 (.02)		<.001	.04 (.02)		.001
	Getting tested	N/A	N/A	.03 (.02)		.004	.11 (.02)		<.001
	Learning more information	N/A	N/A	.05 (.01)		<.001	.06 (.01)		<.001
	Other behaviors	N/A	N/A	−.04 (.02)		.01	.03 (.02)		.08
**Health Belief Model variables**									
	Severity	N/A	N/A	N/A		N/A	.10 (.01)		<.001
	Susceptibility	N/A	N/A	N/A		N/A	.20 (.01)		<.001
	Benefits	N/A	N/A	N/A		N/A	−.002 (.01)		.91
Model of *f* values		494.52	<.001	186.74		<.001	207.11		<.001

^a^Model 1 had a change in *R*^2^ of 0.21 and a total *R*^2^ of 0.21.

^b^Model 2 had a change in *R*^2^ of 0.01 and a total *R*^2^ of 0.22.

^c^Model 3 had a change in *R*^2^ of 0.07 and a total *R*^2^ of 0.21.

^d^β is a standardized coefficient.

^e^N/A: not applicable.

**Table 3:** Hierarchical ordinary least squares regression of predictors for the number of favorites (based on Texas public health agencies’ COVID-19–related tweets that were posted between January 1 and June 30, 2020; N=7269).

Variables		Number of favorites					
		Model 1^a^		Model 2^b^		Model 3^c^	
		β^d^ (SE)	*P* value	β (SE)	*P* value	β (SE)	*P* value
**Control**							
	Followers	.50 (.01)	<.001	.50 (.01)	<.001	.55 (.01)	<.001
**Functions**							
	Information	.05 (.03)	<.001	.05 (.03)	<.001	.01 (.03)	.31
	Action	.09 (.01)	<.001	.09 (.02)	<.001	.11 (.02)	<.001
	Community	.08 (.02)	<.001	.08 (.02)	<.001	.09 (.02)	<.001
**Types of actions proposed**							
	Handwashing	N/A^e^	N/A	−.03 (.03)	.049	−.06 (.03)	<.001
	Social distancing	N/A	N/A	.03 (.03)	.03	.03 (.03)	.04
	Mask wearing or face covering	N/A	N/A	−.001 (.03)	.91	.03 (.03)	.12
	Staying at home	N/A	N/A	.04 (.02)	<.001	.04 (.02)	.004
	Getting tested	N/A	N/A	−.01 (.02)	.58	.05 (.02)	<.001
	Learning more information	N/A	N/A	−.01 (.01)	.64	.01 (.01)	.56
	Other behaviors	N/A	N/A	−.03 (.02)	.07	.02 (.02)	.14
**Health Belief Model variables**							
	Severity	N/A	N/A	N/A	N/A	.04 (.01)	.008
	Susceptibility	N/A	N/A	N/A	N/A	.19 (.01)	<.001
	Benefits	N/A	N/A	N/A	N/A	−.01 (.01)	.62
Model of *f* values		707.52	<.001	260.29	<.001	243.51	<.001

^a^Model 1 had a change in *R*^2^ of 0.28 and a total *R*^2^ of 0.28.

^b^Model 2 had a change in *R*^2^ of 0.003 and a total *R*^2^ of 0.28.

^c^Model 3 had a change in *R*^2^ of 0.04 and a total *R*^2^ of 0.32.

^d^β is a standardized coefficient.

^e^N/A: not applicable.

In terms of promoting public sharing or retweeting behaviors, tweets that fulfilled the information and action functions were more likely to be retweeted. Tweets that contained mentions of covering one’s face, sheltering in place, getting tested, and seeking COVID-19–related information were also more likely to be retweeted, whereas those containing handwashing information were significantly less likely to be retweeted (*P*<.001). Additionally, severity and susceptibility significantly promoted retweeting tendencies (severity: *P*<.001; susceptibility: *P*<.001).

In terms of predicting the number of favorites that tweets received, the results showed slightly different patterns. Tweets that were primarily about promoting action and building community were more likely to receive favorites from the public compared to those about providing information. Furthermore, content that included information about social distancing, sheltering in place, and getting tested were more likely to be favorited, whereas tweets that mentioned handwashing behaviors had a consistently low chance of being favorited by the public. Consistent with the other engagement indicator, the severity and susceptibility health beliefs also significantly predicted the chance of being favorited by the public (severity: *P*=.008; susceptibility: *P*<.001).

## Discussion

### Principal Findings

Governmental agencies are among the most trusted sources of COVID-19–related information [31]. Public health agencies shoulder the responsibility of promptly providing locally relevant pandemic updates, prevention guidelines, and relevant policies to the public during the COVID-19 pandemic. This study uses the HBM and examines social media functions to understand how public health agencies in Texas communicate COVID-19 pandemic–related information to the public via Twitter and assesses the empirical relationships between various message features and social media engagement outcomes. We found that public health agencies mostly used Twitter to share information and they used Twitter to promote action and community with less frequency. Tweets that served the action function were the most likely to be retweeted and liked. Beliefs about susceptibility, severity, and benefits were the most frequently covered health beliefs. Tweets that provided susceptibility and severity information resulted in more public engagement in terms of the number of retweets and endorsements.

The information function was the most prominent function among the studied tweets, followed by the action and community functions. This is consistent with the findings of an earlier study that examined the tweets of Canadian public health agencies [32]. Information is of paramount importance to the public, especially during the early stages of an infectious disease outbreak, which are characterized by a lack of information and a high level of uncertainty. In terms of public engagement, tweets that served the information function were more likely to be retweeted. An existing study with a smaller sample size than that of our study has also shown that science-based tweets about COVID-19 are more likely to be retweeted than tweets that contain false information [33]. This means that useful information can be further disseminated through retweeting. Furthermore, tweets that promoted different preventive measures were the most likely to be retweeted and liked, which shows that the Twitter users are spreading such recommendations through retweeting. Finally, retweets that served the action and community functions were more likely to be liked. This means that readers tend to respond favorably to such tweets to show their support.

Although the HBM has been traditionally used to study psychological predictors of individuals’ adoption of preventative behaviors, it was used in this study to examine the public’s collective responses to health messages in terms of public engagement. Beliefs about susceptibility, severity, and benefits were the most frequently covered health beliefs, whereas information about barriers and self-efficacy was absent from most tweets. This means that communicating the risks of COVID-19 to the public was the priority of Texas public health agencies. Emphasizing health benefits is conducive to the adoption of preventative behaviors [11]. In addition, we found that tweets containing beliefs about susceptibility often resulted in more public engagement in terms of the number of favorites and retweets, whereas the benefits of prevention methods did not increase public engagement. It appears that the public is more interested in learning about the risks of COVID-19 than in learning about preventive behaviors during the early stage of a public health crisis, as the Crisis and Emergency Risk Communication Model has indicated previously [34]. Although this study focused on how message characteristics affect public engagement, other research has shown that public health agencies’ positions in a network (eg, whether the organization occupies a “star” position, which represents their network centrality) also affected the two-way communication between agencies and the public [35].

### Public Health Implications

Our findings identified several strategies that public agencies could adopt to more effectively communicate risk information during an unfolding pandemic. First, the fact that informative tweets were more likely to be retweeted suggests that public agencies should continue to use Twitter as an information dissemination tool to increase their community outreach efforts. The sharing and retweeting function of social media can allow public health agencies to disseminate timely, credible, and easy-to-share information at a large scale, which directly and indirectly helps combat health misinformation [21]. Furthermore, as action-oriented messages were more likely to be favored, public agencies should consider incorporating specific action items into their tweets. In other words, the public needs not only factual information about the pandemic but also specific guidance and concrete action items, which can further boost the public support of public agencies.

Second, although emphasizing the susceptibility and severity of the disease increased public engagement, directly communicating the benefits of preventive behaviors was less effective in promoting public engagement. Given the importance of educating the public about prevention behaviors for infectious diseases, public agencies need to be more creative when designing, framing, and implementing social media messages about preventive behaviors. Furthermore, self-efficacy information was almost completely absent from the tweets of public health agencies. Telling the public that they are capable of performing a recommended behavior is essential in increasing the adoption of such behaviors.

### Methodological Implications

In terms of methodology, this study demonstrates the feasibility of using natural language processing to identify theoretical constructs such as social media functions and health beliefs. We showed that a relatively small training data set could be used to create algorithms for the classification of a much larger corpus of Twitter data. The method established in this study can be easily used to classify COVID-19–related tweets according to different types of organizations (eg, hospitals, community organizations, and media) and individuals (eg, politicians and physicians) in and beyond the state of Texas.

### Limitations and Directions for Future Research

This study only examined public health agencies’ tweets from a single state in the United States, and our data only covered the first wave of the COVID-19 outbreak in the United States. According to the Crisis and Emergency Risk Communication Model, the public has different informational and emotional needs during different stages of an outbreak [34]. It is important to examine agencies’ Twitter content during the later stages of the outbreak. Fortunately, our research method can be easily and longitudinally scaled to study more Twitter content from different parts of the United States. Future studies may examine how message features may vary across different stages of the pandemic and how their resulting public engagement outcomes shift over time. We only examined the text of tweets but did not examine pictures and videos. Future studies should examine how pictures or videos affect public engagement. Additionally, in terms of the communication functions of governmental organizations, an earlier study has suggested that their communication efforts are often fragmented; there is a lack of Twitter mentions, coordination, and mutual retweets among different governmental organizations [36]. Future research could examine the coordination and inconsistency among public health agencies at the local, state, national, and international levels. This approach was piloted in a recent study [37].

### Conclusions

This study examines the content of COVID-19–related tweets that were published by the public health agencies in Texas during the first 6 months of 2020. We found that although public health agencies mostly used Twitter to disseminate pandemic-related information, they could use the Twitter platform to further promote preventative actions, since in this study, the public positively responded to tweets that promoted actions. Furthermore, the public was most likely to engage with tweets that described people’s susceptibility to contracting COVID-19, as such information helped them to understand the risk of the disease. However, there was a lack of information that convinced the public of the high feasibility of proposed preventative behaviors and increased the public’s confidence. Public health agencies can vastly expand their reach during public health crises by steadily building up their follower bases.

## Abbreviations

BERT: bidirectional encoder representations from transformers
HBM: Health Belief Model
OEM: Office of Emergency Management
RQ: research question
